# Editorial: Molecular Physiology in Molluscs, Volume II

**DOI:** 10.3389/fphys.2022.929931

**Published:** 2022-06-17

**Authors:** Xuekai Zhang, Youji Wang, Xiaotong Wang

**Affiliations:** ^1^ School of Agriculture, Ludong University, Yantai, China; ^2^ International Research Center for Marine Biosciences, Ministry of Science and Technology, Shanghai Ocean University, Shanghai, China

**Keywords:** molluscs, molecular physiology, transcriptomics, phenotypic plasticity, toxicology, innate immune, gene interaction, molecular transport

Physiology applies the basic rules of chemistry, physics, and biology to study the activities of organisms, including the study of the regulation of the synthesis of biomolecules at the molecular level, and the details of providing nutrients to support mitochondrial metabolism at the subcellular level (Carroll, 2007). Molecular physiology focuses on molecules and continues through the interactions of an organism with its environment. Molluscs have played an important role in the history of physiology, and two groups of scientists studying mollusc physiology shared the Nobel Prize in Physiology or Medicine in 1963 and 2000, respectively (Wilbur and Yonge, 1964; Kandel, 2005; Wang and Wang, 2019). Despite these achievements, there is still considerable room for further studies on phenotypic plasticity, toxicology, innate immunity, gene interaction, and molecular transport in molluscs. Fortunately, the application of advanced techniques over the past decade has greatly promoted the progress in molecular physiology of molluscs. Therefore, the Frontiers editorial office had suggested that we further organize and publish the Research Topic of molecular physiology in molluscs in *Frontiers in Physiology*. Finally, 25 articles were published and summarized in the *Molecular Physiology in Molluscs, Volume II* in *Frontiers in Physiology*, which covers a rich and varied range of topics, with an emphasis on the molecular mechanisms of molluscan physiology.

Hybridization is an important process of intraspecific and interspecific gene exchange, often accompanied by heterosis or inferiority, as an effective breeding method, and heterosis and phenotypic plasticity are considered basic prerequisites for hybrid breeding (Newkirk and Haley, 1983; Gutierrez, et al., 2018; Meng, et al., 2021). While the molecular basis of heterosis in molluscs remains unclear, Liang et al. deployed interbreeding between two geographic genotypes of *Haliotis diversicolor* and confirmed the presence of non-additive, dominant, and over-dominant models in the transcriptome and miRNAome, of which facilitate the advantageous integration of the parental alleles into the hybrid, thereby contributing to heterosis. Wang et al. identified growth heterosis of the hybrids and co-dominant expression of color genes in the mantle, revealing the formation and evolution of the mantle color polymorphism of *T. crocea*.

Phenotypic plasticity is an adaptive mechanism involving the ability of organisms to assume different phenotypes without genotype changes (Pfennig, et al., 2010). Species differ in their ability to adapt to the environment, and the plasticity of environmental adaptation is not only related to their own genetics, such as gene plasticity, but may also be closely related to the interaction between intestinal flora and the environment. Huang et al. found that intragenic methylation can regulate growth, development, transduction, and apoptosis in *H. discus hannai*, offering a new perspective of the epigenetic mechanism underlying phenotypic plasticity in climate change adaptation for marine organisms. Coincidentally, Christopher et al. highlighted that transcriptome plasticity allows highly resilient organisms such as *Crepidula fornicata* to acclimate to ocean acidification. Lai et al. identified the *PmPIAS* gene in *Pinctada fucata martensii* and list 11 cold tolerance-related SNPs among different genotypes and alleles that provide a potential molecular marker for selective breeding of cold tolerance. Zheng et al. investigated the existing bacterial communities in *P. f. martensii* intestine and the surrounding water from two sites using 16S rRNA-based sequencing and demonstrated the plasticity of intestinal flora to the environment. Given the limited research and the importance of intestinal microbial communities, this study presented pivotal background information on the prevention of diseases caused by aquatic microorganisms.

Bivalves are an important source of high-quality protein for humans, and bivalve toxicology has always been a hot area of concern due to food safety issues. Without a doubt, heavy metal poisoning and ammonia are two major threats to benthic molluscs, Wang et al. found that Cu^2+^ can significantly inactivate the peroxidase and antibacterial activity of homodimer hemoglobin of *Tegillarca granosa* by disrupting its heme pocket structure, which is a non-competitive mode of inhibition. Sun et al. analyzed the association between the polymorphism of glutamate dehydrogenase gene (GDH) and ammonia tolerance, and listed two significantly related SNPs of GDH with ammonia tolerance in *Sinonosinula constricta*, speculating that the GDH may play an important role in the defense against ammonia. In addition to heavy metal poisoning, harmful algal blooms and organic pollution have been commonly found in various water bodies and persist for longer periods than in the past (Brown, et al., 2020; Griffith and Gobler, 2020). Okadaic acid (OA) and paralytic shellfish poisoning (PSP) are two major poisoning toxins of toxic dinoflagellates, one is diarrhetic and the other is neuroparalytic for humans. Wang et al. analyzed the changes in oxidative stress biomarkers, detoxification genes, and proteomics in *Chlamys farreri*, and revealed the mechanisms of toxicity when exposed to OA. Zhu et al. found 14 *PyIAP*s significant responses in the hepatopancreas and kidneys of *Patinopecten yessoensis* when exposed to PST-producing dinoflagellates, and more than 85% of these genes are from the expanded subfamilies BIRC4 and BIRC5, implying the adaptive expansion of bivalve IAPs to algae biotoxins.

Due to the presence of blood sinuses, most molluscs have an open circulatory system. Therefore, discussion on innate immunity has always been important, especially in the study of blood and serum of bivalves (Farabegoli, et al., 2018; Campos, et al., 2020). Defensins have been isolated from metazoans, and as an important component of innate immunity, their role in cellular immunity remains unanswered. Han et al. found that the hemocytes of *Ruditapes philippinarum* can release defensin under *vibrio* stress, and demonstrated their involvement in extracellular traps. Polyamine putrescine (Put) is a small cationic amine that plays an essential role in controlling the innate immune response, but has been poorly studied in molluscs. Cao et al. demonstrated that the content of Put increased in the serum of *P. f. martensii* after lipopolysaccharide (LPS) stimulation, followed by weakened oxidative stress and hemocyte autophagy, indicating that Put may have anti-inflammatory function in molluscs. Rojas et al. revealed that the veliger larvae of *Argopecten purpuratus* exhibit the lowest metabolic capacity when challenged by bacteria, during which they would have a reduced capacity to express immune genes, resulting in a higher susceptibility to pathogen infections.

Giant clams harbor a large amount of phototrophic symbiodiniaceae dinoflagellates, providing them with photosynthate and amino acids which, in turn, need to supply them with inorganic carbon and nitrogen. The unique mechanism of molecular transport between *Tridacna squamosa* and symbiotic dinoflagellates has attracted considerable attention. Pang et al. cloned and reported three major sequences of the nitrate transporter 2 (NRT2) derived from *T. squamosa*, and proved that different symbiotic dinoflagellates may have different NO_3_^−^ transport potentials using NRT2 as molecular indicators for the first time. Ip et al. elucidated the possible functions of the sodium-dependent phosphate transporter protein 1-homolog that it may co-transport Na^+^ and H_2_PO_4_^−^, and pointed out that accumulation of Pi in the kidney of *T. squamosa* may be unrelated to the restriction of the availability of Pi to the symbionts to regulate their population. Pang et al. found that the expression level of ammonium transporter 2 in symbiotic dinoflagellates of *T. squamosa* increased during illumination, suggesting that the coccoid dinoflagellates residing in the outer mantle could augment the potential for ammonia absorption in alignment with photosynthesis, as the assimilation of ammonia required an increase in the supply of carbon chains. These results provide us with a clear view of nutrient transport in a clam-algal symbiotic system.

Gene regulation is an important molecular physiological process in molluscs, particularly in neuroendocrine regulation, growth, and sex determination. Yang et al. revealed a close relationship between dietary habit transition and metamorphosis, and identified the developmental process of the neuroendocrine system of *Rapana venosa*, providing insight into the process of metamorphosis in carnivorous gastropods. Retinoic acid (RA) is an important hormone that plays a crucial role in the regulation of developmental and differentiation processes in which RA-activated responses are mediated by the RA receptors (RARs) and the retinoid X receptors (RXRs) in vertebrates (Balmer and Blomhoff, 2002; Ghyselinck and Duester, 2019). Jin et al. found that RAR is localized in the nucleus and highly expressed in gonads and hemocytes of *Crassostrea gigas*, and CgRAR and can interact with CgRXR to form a heterodimeric complex and function during the developmental process. The testis-specific serine/threonine kinase (Tssk) family plays a key role in vertebrate spermatogenesis, but investigations in molluscs are still lagging. Xue et al. identified five members of the Tssk family in *Argopecten irradians*, including a new Tssk7 that had never been reported before, and all five Tssks were localized in the spermatids and spermatozoa. Research into the function of the Tssk family may contribute to a better understanding of sperm development in molluscs and may contribute to the knowledge of male sterility in some bivalves. Wang et al. studied the sex-determiner transformer-2 homologue in *Hyriopsis cumingii*, and found the probe signal of Hctra-2 mRNA in both male and female gonads, indicating that Hctra-2 was involved in gonadal development. In addition, they also speculated a cascade regulatory relationship between two sex-related genes (Hcfem-1b and Hcdmrt) and Hctra-2, but with different modes of action in males and females.

Given their ecological importance, the biological metabolism and mineralization of molluscs have always been the focus of molluscan research. Brood care is a cephalopod-specific behavior that can significantly improve the survival rate and constitution of larvae, but its metabolism mechanism is rarely studied. Bao et al. provided a core set of metabolic genes of the brood-care behavior, and attempted to explain the mechanism by comparing the transcriptome profiles of *Octopus ocellatus* larvae at different time points. *Charonia tritonis* is an endangered gastropod species of ecological and economic importance, but there are few studies on this species. Zhang et al. compared the transcriptome profiles between the foot muscle and mantle tissue of *C. tritonis*, and highlighted the biomineralization-related process to promote the understanding of the ecological mechanisms of biomineralization in gastropoda.

Oysters are globally cultivated bivalves; *Crassostrea sikamea* and *C. angulata* are sympatric oysters, but each has its own flavor. At the same time, data on differences in their nutrition and taste are lacking. Liu et al. compared the molecular basis of taste and micronutrient content of the two species, and pointed out that *C. sikamea* contains abundant glycogen, Ca, Zn, and Cu, as well as volatile organic compounds, especially aldehydes, which may contribute to their special taste rather than free amino acids and flavor nucleotides. Chromosome manipulation in marine bivalves has received considerable attention due to the great success of triploid oysters (Stanley, et al., 1981; Yang, et al., 2000; McCombie, et al., 2005), whereas the mechanism of chromosome segregation following Cytochalasin B (CB) treatment inhibition has not been well studied. Li et al. first proposed that chromosome segregation during meiosis II may be determined by the spindle patterns and heavily influenced by the number of centrosomes in *Chlamys farreri* eggs. In addition, Long et al. obtained five pure zooxanthellae species from antler coral by molecular identification, and analyzed their effects on larval metamorphosis and progeny performance of *Tridacna squamosa* and *T. crocea*. The results suggested that all these zooxanthellae species can supply nutrition through photosynthesis, and the growth characteristics of its progeny are most affected by species, followed by the zooxanthellae species.

In summary, the articles in this Research Topic presented recent advances in the field of molecular physiology in molluscs that will benefit molluscs physiology science for many years to come.
